# Aortic response to cancer therapy: a narrative review of the radiotherapy paradox and clinical implications

**DOI:** 10.1186/s43044-026-00740-9

**Published:** 2026-04-29

**Authors:** Bibin Joseph, P. Ajay Chand

**Affiliations:** 1 Faculty of Life and Allied Health Sciences, M S Ramaiah University of Applied Sciences, Bengaluru, India; 2 Department of Radio-Diagnosis, Father muller college of allied health sciences, Mangalore, India

**Keywords:** Aorta, Aneurysm, Dilatation, Chemotherapy, Radiotherapy, Computed tomography, Opportunistic screening, Vascular toxicity, Aortic pathology

## Abstract

**Background:**

Cancer treatments such as chemotherapy and radiotherapy significantly impact the cardiovascular system. While cardiac toxicity is well characterized, the effects on the aorta, the body’s largest artery, remain less understood. Given that aortic weakening may lead to life-threatening conditions such as aneurysm formation and rupture, understanding these effects is crucial for improving long-term vascular health in cancer survivors.

**Methods:**

This narrative review synthesizes findings from six selected studies comprising clinical investigations and reviews. It examines the impact of radiotherapy, chemotherapy, cancer-related systemic factors, and incidental aortic findings from radiotherapy planning computed tomography scans, analysing their roles in aortic pathology progression or stabilization.

**Results:**

In this narrative review of six selected studies, radiotherapy was consistently associated with slower aneurysm expansion (~ 1.1 mm/year vs. 2.7 mm/year), suggesting a paradox wherein radiation-induced fibrosis and modulation of inflammatory processes may stabilize the aortic wall. Chemotherapy showed no significant effect on aneurysm growth, with rates comparable between treated patients and controls (~ 2.3 vs. 2.4 mm/year). Cancer-induced sarcopenia and inflammation contributed to adverse aortic remodelling within one year. Additionally, a high prevalence (9.3%) of thoracic aortic dilatation was identified via opportunistic screening on radiotherapy planning CTs, emphasizing an opportunity for earlier detection and intervention.

**Conclusions:**

The complex interplay between cancer therapies and aortic biology reveals a potentially counterintuitive stabilization of aortic pathology following radiotherapy, in contrast to the neutral effects of chemotherapy and the deleterious influence of cancer-related systemic changes. Radiotherapy planning CT offers an underutilized avenue for vascular screening. Future prospective studies are essential to disentangle these paradoxes and translate mechanistic insights into strategies that protect vascular integrity while optimizing cancer treatment outcomes.

## Background

Cancer therapies like chemotherapy and radiotherapy have notably improved survival rates, but their long-term vascular effects, especially on the aorta, remain less understood. While cardiac complications have received much attention [1], the aorta critical for systemic blood flow has been understudied despite its vulnerability to life-threatening conditions such as aneurysm and dilatation [2, 3]. Radiotherapy’s well known endothelial injury, inflammation, and fibrosis would intuitively suggest exacerbation of aortic pathology [1, 4].

However, emerging data reveal a paradox: radiotherapy appears to slow aneurysm growth in some patients, contradicting expectations of accelerated vascular damage [3]. This paradox raises fundamental questions about radiation-induced vascular biology. Chemotherapy’s effects are less clear but seem neutral regarding aneurysm progression [2], while systemic cancer effects such as sarcopenia promote adverse remodelling independent of direct treatment [5].

This narrative review synthesizes recent studies exploring these conflicting reports and biological mechanisms. By focusing on the paradoxical role of radiotherapy, it aims to illuminate complexities in aortic responses that have important clinical ramifications, particularly for surveillance, intervention, and surgical management [6].

## Methodology

Purpose of the Review: This narrative review aims to synthesize the emerging evidence on the effects of cancer therapies like chemotherapy and radiotherapy on the aorta, with particular emphasis on the counterintuitive observation that radiotherapy may slow aneurysm growth the “radiotherapy paradox”, the neutral effect of chemotherapy, the role of cancer related systemic factors such as sarcopenia, and the potential for opportunistic screening using radiotherapy planning CT scans.

The search was conducted in 20 December 2025 and covered publications from January 2010 to November 2025. The following search string was used (with appropriate Boolean operators and database-specific adjustments):

Search String: (aortic aneurysm OR aortic dilatation) AND (“radiation therapy” OR “cancer therapy”) AND (growth OR expansion OR remodelling OR dilatation).

Data Bases: PubMed, Scopus, Research Scholar, Google Scholar.

Six studies were ultimately selected based on the following.

Inclusion criteria:

1. Clinical studies (retrospective cohorts, longitudinal imaging studies, or cross-sectional analyses) and targeted reviews direct focus on the effects of chemotherapy and radiotherapy on aortic pathology (aneurysm growth, dilatation, or remodelling).
2. Availability of quantitative aortic outcomes (e.g., growth rates or prevalence of dilatation).
3. Publication in English.

Exclusion criteria:

1. Included purely preclinical studies without clinical correlation, studies focused solely on cardiac toxicity without aortic assessment, case reports, and editorials.
2. The final selection prioritized studies with high clinical relevance, methodological rigor, and direct contribution to understanding the radiotherapy paradox.
3. Although a formal PRISMA protocol was not followed (as this is a narrative review), the selection process aimed to capture the most representative and recent evidence in this emerging field.

### Selection flow chart


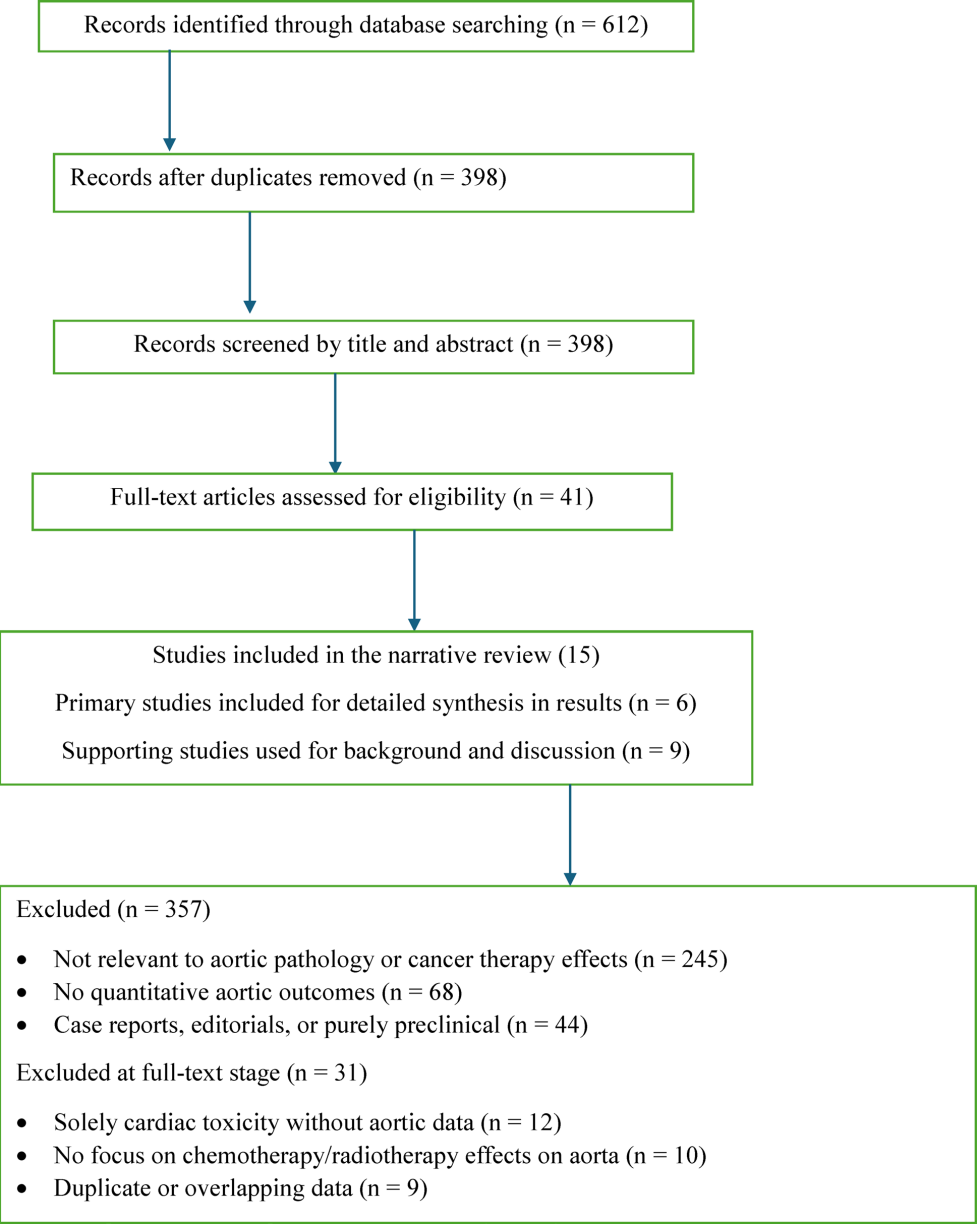


The deliberate choice of a limited number of studies was intended to ensure a focused and clinically meaningful synthesis and to highlight novel and paradoxical findings, particularly the slowing of aneurysm growth after radiotherapy exposure. However, this selection process is inherently subjective and non exhaustive, and thus results should be interpreted cautiously as hypotheses-generating rather than definitive conclusions.

Summary and Limitations: Due to the budding stage of research in this area, only a small number of relevant clinical studies were available. This review is therefore inherently subjective and non-exhaustive. All interpretations and proposed mechanisms should be viewed as hypothesis-generating rather than conclusive. Prospective studies with larger cohorts and longer follow-up are essential to validate these observations.

## Review of articles

This review brings together six studies, each exploring the connection between cancer treatment and the aorta in different ways and followed a literature review method.

- Article 1 (Martin et al.): This study used a retrospective cohort design to investigate whether chemotherapy altered the natural history of aortic aneurysms. Patients with malignancy and known aortic aneurysms who received chemotherapy were identified, and their outcomes were compared with those of a surveillance cohort with aneurysms but no chemotherapy exposure. Aneurysm growth was assessed using serial computed tomography or magnetic resonance imaging, and growth rates were expressed in millimetres per year. In addition to tracking changes in aneurysm diameter, the study recorded aneurysm related events such as rupture and the need for elective or urgent repair [1]. Strengths: Direct comparison with a non-chemotherapy control group. Limitations: Retrospective design, potential confounding by cancer type and comorbidities (e.g., hypertension, smoking), and heterogeneity in chemotherapy regimens.
- Article 2 (Becker von Rose et al.): This study also employed a retrospective cohort methodology, but their focus was on patients with abdominal aortic aneurysm who underwent radiotherapy. The study included cancer patients with abdominal aortic aneurysm exposed to radiotherapy, and these were compared with both cancer patients who did not undergo radiotherapy and with a noncancer reference cohort with abdominal aortic aneurysm. radiotherapy treatment plans were carefully reviewed to determine the radiation dose received by the aneurysm, allowing the investigators to evaluate whether in-field or out-of-field exposure affected growth. Aneurysm diameters were measured from imaging before and after Radiotherapy, and the primary outcome was annual growth rate [2]. Strengths: Dose assessment (in-field vs. out-of-field). Limitations: Retrospective, relatively small sample, possible confounding by cancer-related factors.
- Article 3 (Groarke et al.): Conducted a review summarizing the cardiovascular complications of thoracic Radiotherapy. Their methodology involved synthesizing existing clinical and experimental literature on the subject, with specific emphasis on large vessel vasculopathy, including effects on the aorta [3]. Narrative review which inherently limited by the quality of included primary studies.
- Article 4 (Igić): This review focused on radiotherapy and abdominal aortic aneurysm. This review brought together available evidence on epidemiology, potential mechanisms such as endothelial injury and fibrosis, and clinical observations suggesting that radiotherapy may influence aneurysm behaviour [4]. Narrative review which inherently limited by the quality of included primary studies.
- Article 5 (Gao et al.): This one year longitudinal computed tomography study of 100 cancer patients. Measured aortic diameters and tortuosity; correlated with skeletal muscle mass and inflammatory biomarkers adopted a longitudinal imaging design. The investigators retrospectively analysed 100 cancer patients who had undergone contrast enhanced computed tomography scans at baseline and again after one year. They measured aortic diameters and tortuosity across multiple regions and quantified changes over time. Importantly, these vascular measures were correlated with skeletal muscle mass derived from the same computed tomography scans, as well as with inflammatory biomarkers including C-reactive protein and lipid profiles. Statistical modelling was used to test the independent associations between muscle loss and aortic remodelling [5]. Strengths: Longitudinal design with paired CT scans. Limitations: Single-center, modest sample size (*n* = 100), short follow-up (1 year). The anatomical level used for skeletal muscle quantification was not specified in the study, limiting reproducibility and comparison with established sarcopenia assessment protocols. Additionally, inter-observer variability in aortic diameter measurements was not reported, which is relevant when interpreting small annual changes.
- Article 6 (Lattu et al.): Cross-sectional study of 861 women undergoing Radiotherapy -planning computed tomography for breast cancer. Reported prevalence and risk factors of thoracic aortic dilatation conducted a large cross sectional analysis of 861 women with breast cancer who underwent non contrast computed tomography scans as part of adjuvant Radiotherapy planning. The scans were used to measure thoracic aortic diameters at predefined anatomical sites. Aortic dilatation was defined as a diameter greater than 40 mm, following European Society of Cardiology guidelines. The prevalence of thoracic aortic dilatation (TAD) was reported, and associations with clinical risk factors, including age, hypertension, prior cerebrovascular disease, and aortic valve pathology, were examined [6]. Strengths: Large sample (*n* = 861). Limitations: Cross sectional (no longitudinal growth data), female only population (breast cancer cohort) and This finding is derived from a female only breast cancer cohort; given the known sexual dimorphism in aortic disease, generalizability to male patients or other cancer types requires caution.

## Results

### Summary of chemotherapy studies

Chemotherapy did not significantly alter aneurysm growth rates compared with controls (2.3 mm/year vs. 2.4 mm/year) [2]. Only two patients experienced rupture requiring immediate intervention. These findings contrast with isolated case reports of rapid expansion during chemotherapy. Chemotherapy, as a broad category, did not significantly alter aneurysm growth rates compared with controls (2.3 mm/year vs. 2.4 mm/year) in the evaluated cohort [2]. Only two patients experienced rupture requiring immediate intervention. These findings contrast with isolated case reports of rapid expansion during chemotherapy. However, chemotherapy encompasses heterogeneous drug classes with differing vascular toxicities. Agents such as VEGF pathway inhibitors (e.g., bevacizumab, sunitinib) have been associated with increased risk of aortic dissection and aneurysm formation, while antimetabolites may accelerate growth in some subgroups. The neutral overall effect observed may reflect short follow-up durations or predominance of regimens with lower vascular toxicity in the studied population. Longer term data and subgroup analyses by drug class are needed before concluding broad neutrality [7, 8]. Additionally, the duration, cumulative exposure, and specific treatment regimens of chemotherapy were not consistently analysed across studies, which may influence long-term vascular outcomes and limit the interpretation of a neutral effect.

### Summary of radiotherapy studies

In patients with abdominal aortic aneurysm undergoing radiotherapy, annual growth rates were significantly lower (~ 1.1 mm/year) compared with non-cancer controls (~ 2.7 mm/year) [2]. Reduced growth was observed in both in-field and out-of-field aneurysms, with no clear dose–response relationship identified.

### Systemic cancer effects

Mechanistic Insights from Review Articles Reviews by Groarke et al. [1] and Igić [4] highlight that radiotherapy induces endothelial injury, medial fibrosis, and accelerated atherosclerosis. While these changes are generally considered detrimental to vascular health, they may exert contrasting effects in the context of aneurysmal disease, however, the one-year follow-up period is relatively short for a chronic process such as aortic remodelling. In the one-year longitudinal CT study of 100 cancer patients, increased aortic diameter and tortuosity were associated with skeletal muscle loss that is sarcopenia and elevated inflammatory markers [5]. This association likely reflects a shared systemic pro-inflammatory state (cancer cachexia) rather than direct causation from sarcopenia to aortic remodelling. Both phenomena may be driven by chronic inflammation and elevated cytokines that promote proteolytic degradation of the aortic matrix.

### Opportunistic CT screening findings

Opportunistic Screening Findings In a cross sectional analysis of 861 women undergoing radiotherapy planning CT for breast cancer, the prevalence of thoracic aortic dilatation (> 40 mm) was 9.3% [6]. Independent risk factors included older age, hypertension, prior cerebrovascular disease, and aortic valve pathology. While the 9.3% prevalence of thoracic aortic dilatation supports opportunistic screening on radiotherapy planning CTs, potential risks of overdiagnosis, patient anxiety, and the burden of lifelong surveillance in cancer patients (whose primary priority is oncologic treatment) should be considered. Clear referral algorithms and risk-benefit discussions are needed before widespread implementation.

### Comparison of clinical research studies

See Table 1.

**Table 1:** Comparison of different articles on effects of chemotherapy and radiotherapy on Aorta

Article	Design	Population	Methods	Main findings
1. Martin et al. [2]	Retrospective cohort	Patients with malignancy and known aortic aneurysm receiving chemotherapy; compared with surveillance cohort	Serial CT/MR imaging, growth rates calculated, aneurysm events tracked	Chemotherapy did not accelerate aneurysm growth (2.3 vs. 2.4 mm/year). Only 2 ruptures reported.
2. Becker von Rose et al. [3]	Retrospective cohort	Cancer patients with AAA who underwent RT; compared with non-RT cancer patients and non-cancer controls	Imaging before/after RT; annual growth calculated; RT dose assessed	RT patients had slower AAA growth (~ 1.1 mm/year) vs. non-cancer (~ 2.7 mm/year). No dose–response.
3. Gao et al. [5]	Longitudinal imaging study	100 cancer patients with baseline and 1-year follow-up CT	Aortic diameters and tortuosity measured; correlated with skeletal muscle and biomarkers	Aortic dilatation and tortuosity worsened with sarcopenia. Muscle loss independently predicted remodelling.
4. Lattu et al. [6]	Cross-sectional prevalence study	861 breast cancer patients undergoing RT planning CT	Outer-to-outer aortic diameters measured; dilatation defined as > 40 mm	9.3% prevalence of thoracic aortic dilatation; age, hypertension, cerebrovascular and valve disease were key risk factors.

## Discussion

Given the retrospective nature and limited number of studies, these findings should be considered preliminary and hypothesis-generating. The relationship between cancer therapies and the aorta is intricate, involving multiple factors and sometimes surprising outcomes. Notably, several recent studies demonstrate a paradoxical observation that radiotherapy, despite its known vascular toxicity, may be linked with slower aneurysm growth [1, 3, 4]. Chemotherapy appears largely neutral concerning aneurysm progression [2], whereas cancer itself may drive adverse vascular remodelling through mechanisms such as sarcopenia and inflammation [5]. Moreover, radiotherapy planning computed tomography scans have revealed a significant prevalence of thoracic aortic dilatation, presenting an opportunity for opportunistic clinical screening [6]. Together, these findings underscore the necessity for deeper mechanistic insight and raise vital clinical considerations, particularly for cardiothoracic surgeons.

### Potential mechanisms of the radiotherapy paradox

The observation that radiotherapy is associated with slower aneurysm growth in selected studies, despite its well documented vascular toxicity, remains incompletely understood and potentially confounded. Several biologically plausible mechanisms have been proposed, although they remain largely hypothetical and require experimental validation:

- Fibrotic scaffolding: Radiation-induced fibrosis and medial scarring may provide mechanical stabilization in some aneurysmal walls.
- Suppression of inflammation: Radiotherapy may reduce chronic inflammatory infiltrates and matrix metalloproteinase activity.
- Vasa vasorum obliteration: While this could theoretically limit inflammatory support and expansion, radiation-induced damage to the vasa vasorum more commonly causes medial ischemia, necrosis, and wall fragility increasing risks of dissection or rupture rather than providing net benefit.
- Modulation of matrix metalloproteinases: Possible direct suppression limiting matrix breakdown.

Importantly, radiotherapy is also known to cause accelerated atherosclerosis, endothelial injury, and loss of vascular elasticity, which can weaken the arterial wall over time. The apparent “stabilization” (1.1 mm/year vs. 2.7 mm/year) may be influenced by confounding factors such as competing mortality from cancer, differences in baseline characteristics, statin/beta-blocker use, or selection bias. No clear dose-response relationship was observed, further questioning a direct causal effect of radiation. Post-radiation tissues are often described as “hostile” for surgery due to dense fibrosis and fragility (“tear like wet paper”), highlighting that any potential benefit on growth rate must be weighed against increased surgical risk and possible long-term fragility [9–11].

### Implications for surgical decision making and patient management

For cardiothoracic surgeons, these paradoxical effects prompt several important clinical questions:

- Surveillance and Intervention Thresholds: If radiotherapy slows aneurysm growth, should surveillance intervals for patients’ post radiotherapy be extended? Could size thresholds for elective repair be reconsidered in this subgroup? These questions necessitate prospective evaluation [12].
- The “Hostile” Irradiated Aorta: Surgery on irradiated tissues is challenging due to dense fibrosis, altered anatomy, and impaired healing [9, 13]. This complexity sometimes favours earlier prophylactic aneurysm repair before radiotherapy in select cases.
- Risk Benefit in Multidisciplinary Care: These findings reinforce the need for collaborative decision making between oncologists, radiation oncologists, and cardiothoracic surgeons. In patients with both cancer and aortic aneurysms, individualized treatment plans must weigh the paradoxical stabilizing effects of radiotherapy against the risks of delayed surgical repair in irradiated tissue [14].
- The hostile irradiated aorta also has important implications for endovascular repair. Endovascular aneurysm repair (EVAR or TEVAR) may be technically more challenging due to dense periaortic fibrosis and pathological stiffening, which can impair stent-graft anchoring and increase the risk of type I endoleaks or graft migration. Although reduced aneurysm growth rates after radiotherapy might delay the need for intervention in selected patients, long-term durability of endografts in irradiated fields remains uncertain because of impaired tissue healing. These considerations highlight the need for careful multidisciplinary assessment when choosing between open surgical repair and endovascular approaches [9, 15].

## Conclusions

**Fig. 1:**
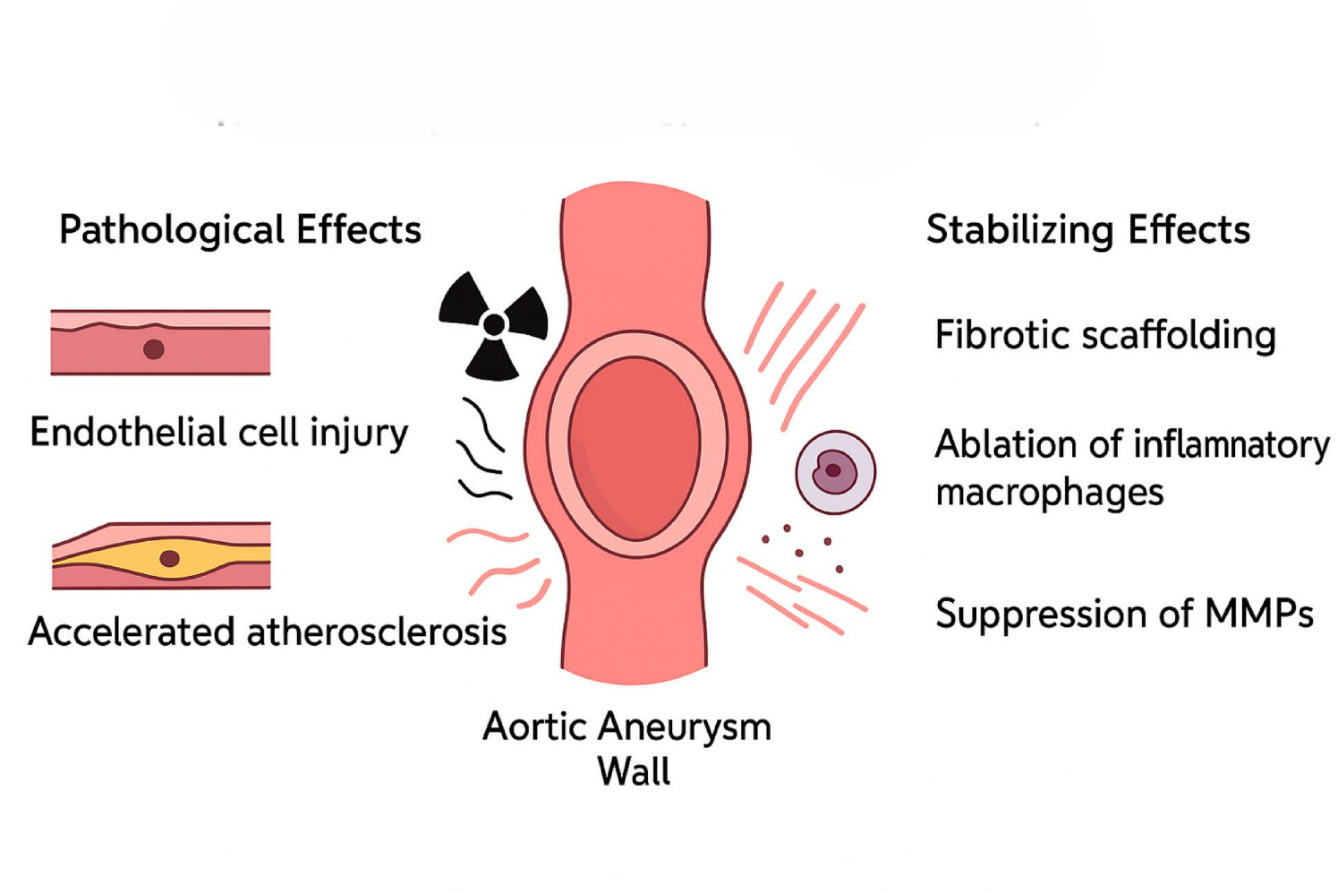
Proposed dual mechanisms of radiotherapy on the aortic aneurysm wall. (The figure is an original illustration generated by using ChatGPT (OpenAI, version 4.o)for this manuscript and does not represent data from any single published study

Chemotherapy does not appear to accelerate aortic aneurysm growth, with multiple studies showing growth rates similar to non-cancer controls [2]. This finding provides reassurance that oncologic treatment can generally be pursued safely in patients with pre-existing aneurysms. In contrast, radiotherapy classically associated with vascular injury, endothelial dysfunction, and fibrosis has been paradoxically linked with slower aneurysm expansion in several cohorts [1, 2, 13]. While counterintuitive, this observation raises the possibility that radiation-induced fibrosis, inflammatory cell depletion, or suppression of matrix metalloproteinases may under certain conditions stabilize the aortic wall rather than weaken it (Fig. 1).

Cancer itself exerts systemic effects that cannot be ignored. Emerging evidence demonstrates that sarcopenia and inflammation in cancer patients promote adverse vascular remodelling within as little as one year [5], suggesting that the malignant state may act as an independent accelerator of aortic pathology. Moreover, the widespread use of radiotherapy planning CT scans has revealed incidental thoracic aortic dilatation in nearly 1 in 10 breast cancer patients [6]. This presents a unique and underutilized opportunity for opportunistic screening and early detection of clinically silent aortic disease.

For cardiothoracic surgeons, these insights have immediate relevance. The paradoxical stabilization of aneurysm growth after radiotherapy raises questions about whether surveillance intervals or size thresholds for intervention should be modified in this subgroup [12]. At the same time, operating on irradiated tissues is notoriously difficult due to fibrosis, poor healing, and distorted anatomy [9, 13], which may favour prophylactic repair before radiotherapy in selected patients. Multidisciplinary discussion involving oncologists, radiation oncologists, and vascular or cardiothoracic surgeons is essential to balance cancer treatment needs with long-term vascular safety [14].

To conclude the relationship between cancer therapy and aortic biology is complex, multifactorial, and sometimes paradoxical. Future research will be crucial not only to resolve these contradictions but also to translate mechanistic insights into novel therapeutic strategies that safeguard both cancer survival and the structural integrity of the great vessels.

## Data Availability

No datasets were generated or analysed during the current study.

## Abbreviations

AAA: Abdominal aortic aneurysm
CT: Computed tomography
MR: Magnetic resonance imaging
RT: Radiotherapy
TAD: Thoracic aortic dilatation
